# Retraction: P311 facilitates the angiogenesis and wound healing function of MSCs by increasing VEGF production

**DOI:** 10.3389/fimmu.2026.1809814

**Published:** 2026-02-20

**Authors:** Frontiers Editorial Office

The journal retracts the 28 January 2022 article cited above.

Following publication, concerns were raised regarding the integrity of the images in the published figures. An investigation was conducted in accordance with Frontiers’ policies and with the collaboration of the authors. It was concluded that the findings and conclusions of the article were unreliable, and the article has been retracted.

This retraction was approved by the Chief Editors of Frontiers in Immunology and the Chief Executive Editor of Frontiers. The authors were informed of this retraction and agree with the decision.

